# Effect of kaempferol on the transgenic Drosophila model of Parkinson’s disease

**DOI:** 10.1038/s41598-020-70236-2

**Published:** 2020-08-14

**Authors:** Rahul, Falaq Naz, Smita Jyoti, Yasir Hasan Siddique

**Affiliations:** Drosophila Transgenic Laboratory, Section of Genetics, Department of Zoology, Faculty of Life Sciences, Aligarh Muslim University, Aligarh, Uttar Pradesh 202002 India

**Keywords:** Drug discovery, Neuroscience

## Abstract

The present study was aimed to study the effect of kaempferol, on the transgenic *Drosophila* model of Parkinson’s disease. Kaempferol was added in the diet at final concentration of 10, 20, 30 and 40 µM and the effect was studied on various cognitive and oxidative stress markers. The results of the study showed that kaempferol, delayed the loss of climbing ability as well as the activity of PD flies in a dose dependent manner compared to unexposed PD flies. A dose-dependent reduction in oxidative stress markers was also observed. Histopathological examination of fly brains using anti-tyrosine hydroxylase immunostaining has revealed a significant dose-dependent increase in the expression of tyrosine hydroxylase in PD flies exposed to kaempferol. Molecular docking results revealed that kaempferol binds to human alpha synuclein at specific sites that might results in the inhibition of alpha synuclein aggregation and prevents the formation of Lewy bodies.

## Introduction

Parkinson's disease (PD) is the second-most common neurodegenerative disorder and about 6.3 million people are affected worldwide. The disease is characterized by four major symptoms tremor, bradykinesia, muscle rigidity, and postural instability. Majority of human PD cases are “sporadic PD” (SPD), because of no genetic linkage, and the remaining cases are “familial PD “(FPD) due to genetic relationships with parents^1^. The pathological hallmarks of PD are the loss of dopaminergic neurons (DA) in the substantia nigra pars compacta (SNpc) region of midbrain and deposition of proteinacious aggregates of α-synuclein, known as “Lewy bodies ” (LB)^2^. Oxidative stress generates reactive oxygen species (ROS), which activates glial cells and activated glial cells generate inflammatory chemokines and cytokines causing mitochondrial dysfunction^3^. Although there are several drugs, for curtailing the symptoms of PD such as DOPA caboxylase, carbidopa; dopamine agonists, such as bromocriptine, pramipexole and ropinirole; MAO-B inhibitors, such as rasagiline and selegiline, but none of them completely cures the disease and are also associated with several side effects^4^. Thus, we need to develop therapies that can target both motor as well as non-motor symptoms and prevents the progression of disease. The animal models are helpful in screening and testing of new therapeutic agents for PD^5^. In this context *Drosophila* has emerged as an effective model for studying PD pathogenesis^6^. Feany and Bender^7^ have developed transgenic *Drosophila melanogaster* for understanding the mechanisms of PD. These PD flies express human α-synuclein, protein aggregation (Lewy bodies formation), decline in dopaminergic neurons and locomotor defects^5^. The brain of *Drosophila* contains clusters of dopaminergic neurons^8^ and these neurons degenerate when the flies express human α-synuclein^9^. The multifunctional attributes of *Drosophila* like robust phenotypes have provided opportunities for genetic and drug screens to identify therapies for PD, and other age-related neurodegenerative disorders^6^.

Kaempferol is a yellow coloured compound commonly found in plants. It has a defensive effect against the brain oxidative damage induced by various types of agents^10,11^. It also exhibit antioxidant potential and is able to cross the blood brain barrier and reduced the neuronal damage^12^. Our present study explores the protective potential of kaempferol in transgenic *Drosophila* model of PD.

## Results

A dose dependent significant increase in scavenging the free radicals was observed at selected doses of kaempferol (Fig. S1; *p* < 0.05). Similarly, the results of the superoxide anion scavenging assay also showed the same pattern. A dose dependent significant increase was observed in scavenging anions by kaempferol (Fig. S2; *p* < 0.05).

The results obtained for the activity of flies are shown in Fig. S3 to S13 (a&b) in the form of activity pattern and chi-square periodogram. The PD flies (Fig. S3a & b) showed a decrease in the activity with an increase in age of the flies compared to the control flies (Fig. S4a & b). The PD flies exposed to 10, 20, 30 and 40 µM of kaempferol showed a dose dependent delay in the loss of activity (Figs. S5a & b–S8a & b). The control flies exposed to 10, 20, 30 and 40 µM of kaempferol showed no difference in the loss of activity Fig. S9 (a & b) to Fig. S12 (a & b). The PD flies exposed to 10^–3^ M of L-dopa also showed a delay in the loss of activity compared to unexposed PD flies (Fig. S13 a & b).

The results obtained for retinal degeneration showed the presence of distorted organization of ommatidia in PD flies (Fig. S14a) in comparison to the control flies (Fig. S14b). The PD flies exposed 10, 20, 30, and 40 µM of kaempferol showed no disorganization in the ommatidia compared to PD flies (Fig S14c to g). The normal flies exposed to 10, 20, 30 and 40 µM of kaempferol and 10^–3^ M of L-dopa also showed no distorted organization of ommatidia (Fig. S14h to k).

**Figure 1:**
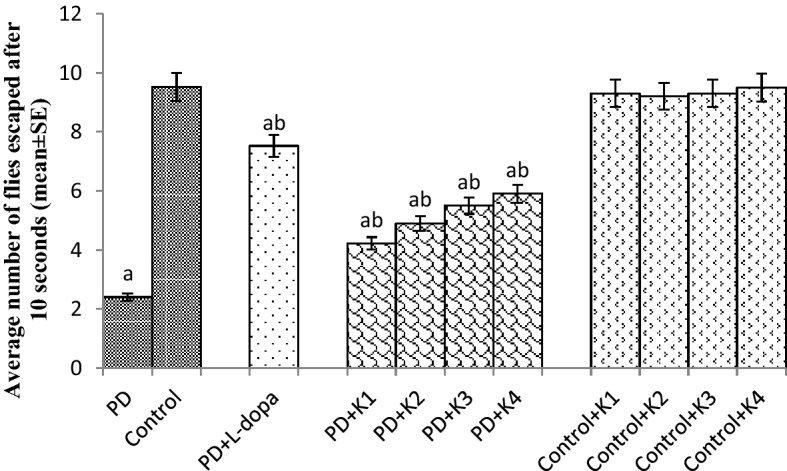
Effect of Kaempferol on the climbing ability. The flies were allowed to feed on the diet supplemented with Kaempferol for 24 days and then assayed for climbing ability. The values are the mean of 5 assays. [^a^significant with respect to control, *p* < 0.05; ^b^significant with respect to PD flies; K = Kaempferol; L-Dopa: 10^−3^ M; K1 = 10 μM; K2 = 20 μM; K3 = 30 μM; K4 = 40 μM; PD: PD flies].

The PD flies showed a significant decrease of 3.95 fold in the climbing ability compared to control flies (Fig. 1; *p* < 0.05). The PD flies exposed to 10, 20, 30 and 40 µM of kaempferol showed a significant delay of 1.75, 2.04, 2.29 and 2.45 folds, respectively, compared to unexposed PD flies (Fig. 1; *p* < 0.05). The control flies exposed to 10, 20, 30, and 40 µM of kaempferol showed no significant difference compared to unexposed control flies (Fig. 1; *p* < 0.05). The PD flies exposed to L-dopa (10^−3^ M) showed a delay of 3.12 fold in the loss of climbing ability (Fig. 1; *p* < 0.05).

**Figure 2:**
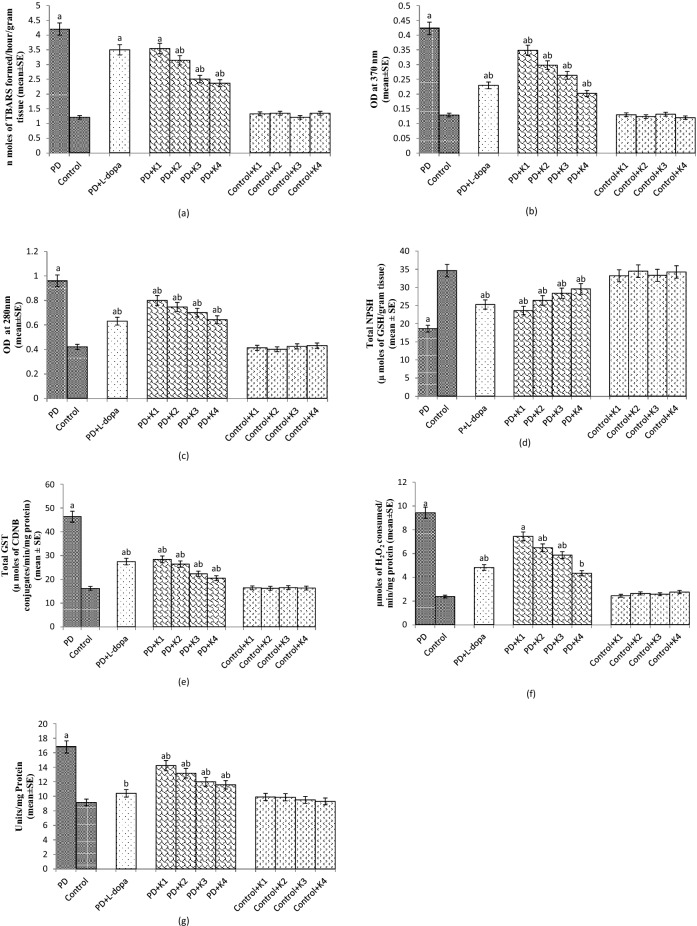
Effect of Kaempferol on TBARS (**a**) protein carbonyl (PC) content (**b**) and monoamine oxidase (MAO) activity (**c**), NPSH content (**d**), Glutathione-S-Transferase (GST) (**e**), catalase (CAT) (**f**) superoxide dismutase activity (SOD) (**g**) measured in the brains of flies. The flies were allowed to feed on the diet supplemented with Kaempferol for 24 days and then assayed. The values are the mean of 5 assays. [^a^significant with respect to control, *p* < 0.05; ^b^significant with respect to PD flies; K = Kaempferol; L-Dopa: 10^−3^ M; K1 = 10 μM; K2 = 20 μM; K3 = 30 μM; K4 = 40 μM; PD: PD flies].

The PD flies showed a significant increase of 3.5 fold in TBARS compared to control flies (Fig. 2a; *p* < 0.05). The PD flies exposed to 20, 30 and 40 µM of kaempferol showed a dose dependent significant decrease of 1.35, 1.68 and 1.82 folds, respectively, in the TBARS compared to unexposed PD flies (Fig. 2a; *p* < 0.05). There was no significant difference in TBARS between the control flies and the control flies exposed to the selected doses of kaempferol (Fig. 2a; *p* < 0.05). The PD flies exposed to L-dopa (10^–3^ M) and 10 μM of kaempferol showed no significant reduction compared to unexposed PD flies (Fig. 2a; *p* < 0.05).

The PD flies showed a significant increase of 3.5 fold in the PC content compared to control flies (Fig. 2b; *p* < 0.05). The PD flies exposed to 10, 20, 30 and 40 µM of kaempferol showed a significant dose dependent decrease of 1.31, 1.40, 1.68 and 2 folds, respectively, in the PC content compared to unexposed PD flies (Fig. 2b; *p* < 0.05). The control flies exposed the selected doses of kaempferol showed no significant difference in the PC content compared to the unexposed control flies (Fig. 2b; *p* < 0.05). The PD flies exposed to L-dopa showed a significant decrease of 1.82 fold in the PC content compared to PD flies (Fig. 2b; *p* < 0.05).

The PD flies showed a significant increase of 2.28 fold in the MAO activity compared to the control flies (Fig. 2c; *p* < 0.05). The PD flies exposed to 10, 20, 30 and 40 µM of kaempferol showed a dose dependent significant increase of 1.2, 1.29, 1.37 and 1.5 folds, respectively, in MAO activity compared to unexposed PD flies (Fig. 2c; *p* < 0.05). The control flies exposed to 10, 20, 30 and 40 µM of kaempferol showed no significant difference in the MAO activity compared to unexposed control flies (Fig. 2c; *p* < 0.05). The PD flies exposed to L-dopa showed a significant decrease of 1.52 fold in the MAO activity compared to PD flies (Fig. 2c; *p* < 0.05).

The PD flies showed a significant decrease of 1.86 fold in the NPSH content compared to control flies (Fig. 2d; *p* < 0.05). The PD flies exposed to 10, 20, 30 and 40 µM of kaempferol showed a significant increase of 1.26, 1.41, 1.52 and 1.59 folds, respectively, in the NPSH content compared to unexposed PD flies (Fig. 2d; *p* < 0.05). The normal flies exposed to 10, 20, 30 and 40 µM of kaempferol showed no significant difference in the NPSH content compared to control flies (Fig. 2d; *p* < 0.05). The PD flies exposed to L-dopa showed a significant increase of 1.35 fold in the NPSH content compared to control flies. (Fig. 2d; *p* < 0.05).

The PD flies showed a significant increase of 2.86 fold in the GST activity compared to control flies (Fig. 2e; *p* < 0.05). The PD flies exposed to 10, 20, 30 and 40 µM of kaempferol showed a dose dependent significant decrease of 1.63 1.75, 2.08 and 2.27 folds, respectively, in GST activity compared to unexposed PD flies (Fig. 2e; *p* < 0.05). The control flies exposed to 10, 20, 30 and 40 µM of kaempferol showed no significant difference in the GST activity compared to unexposed control flies (Fig. 2e; *p* < 0.05). The PD flies exposed to 10^–3^ M of L-dopa showed a significant decrease of 1.69 fold in the GST activity compared to PD flies (Fig. 2e; *p* < 0.05).

The PD flies showed a significant increase of 3.99 folds in the activity of catalase compared to control flies (Fig. 2f; *p* < 0.05). The PD flies exposed to 20, 30 and 40 µM of kaempferol showed a significant decrease of 1.45, 1.60 and 2.17 folds, respectively, in the activity of catalase compared to unexposed PD flies (Fig. 2f; *p* < 0.05). The control flies exposed to 10, 20, 30 and 40 µM of kaempferol showed no significant difference in activity of catalase compared to unexposed control flies (Fig. 2f; *p* < 0.05). The PD flies exposed to 10 μM kaempferol showed no significant decrease in the activity of catalase compared to unexposed PD flies (Fig. 2f; *p* < 0.05). The PD flies exposed to 10^–3^ M of L-dopa showed a significant decrease of 1.96 folds in the catalase activity compared to unexposed PD flies (Fig. 2f; *p* < 0.05).

The PD flies showed a significant increase of 1.83 folds in the activity of SOD compared to control flies (Fig. 2g; *p* < 0.05). The PD flies exposed to 10, 20, 30 and 40 µM of kaempferol showed a dose dependent significant decrease of 1.18, 1.27, 1.40 and 1.44 folds, respectively, in the activity of SOD compared to unexposed PD flies (Fig. 2g; *p* < 0.05). The control flies exposed to 10, 20, 30 and 40 µM of kaempferol showed no significant difference in activity of SOD compared to unexposed control flies (Fig. 2g; *p* < 0.05).The PD flies exposed to 10^–3^ M of L-dopa showed a significant decrease of 1.61 fold in SOD activity compared to unexposed PD flies (Fig. 2g; *p* < 0.05).

**Figure 3:**
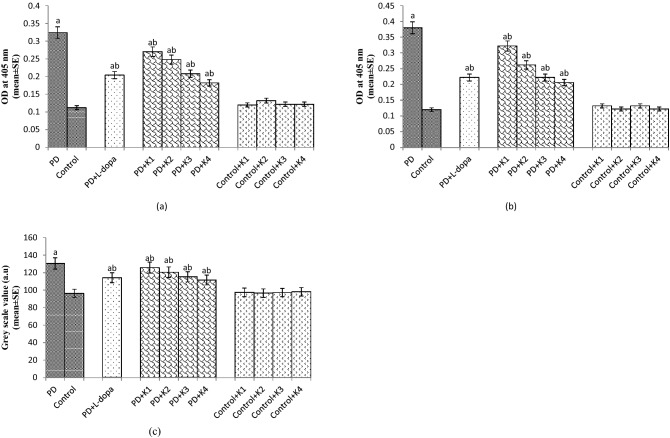
Effect of Kaempferol on Caspase 9 activity (**a**), Caspase 3 activity (**b**) and grey scale value (**c**) measured in the brains of flies. The flies were allowed to feed on the diet supplemented with Kaempferol for 24 days. The values are the mean of 5 assays. [^a^significant with respect to control, *p* < 0.05; ^b^significant with respect to PD flies; L-Dopa: 10^−3^ M; K = Kaempferol; K1 = 10 μM; K2 = 20 μM; K3 = 30 μM; K4 = 40 μM; PD: PD flies].

The PD flies showed an increase of 2.90 fold in the activity of caspase-9 compared to control flies (Fig. 3a; *p* < 0.05). The PD flies exposed to 10, 20, 30 and 40 µM of kaempferol showed a dose dependent significant decrease of 1.18 1.33, 1.60 and 1.77 folds, respectively, in the activity of caspase-9 compared to the PD flies (Fig. 3a; *p* < 0.05). The control flies exposed to 10, 20, 30 and 40 µM of kaempferol showed no significant difference in caspase-9 activity compared to unexposed control flies (Fig. 3a; *p* < 0.05). The PD flies exposed to 10^–3^ M of L-dopa showed a decrease of 1.6 fold in the caspase-9 activity compared to unexposed PD flies (Fig. 3a; *p* < 0.05).

The PD flies showed a significant increase of 3.16 fold in the caspase-3 activity compared to control flies (Fig. 3b; *p* < 0.05). The PD flies exposed to 10, 20, 30 and 40 µM of kaempferol showed a dose dependent significant decrease of 1.18, 1.46, 1.72 and 1.9 folds, respectively, in the activity of caspase-3 (Fig. 3b; *p* < 0.05). The control flies exposed to 10, 20, 30 and 40 µM of kaempferol showed no significant difference in the activity of caspase-3 compared to unexposed control flies (Fig. 3b; *p* < 0.05). The PD flies exposed to 10^–3^ M L-dopa showed a significant decrease of 1.72 fold in the caspase-3 activity compared to unexposed PD flies (Fig. 3b; *p* < 0.05).

The PD flies showed a significant increase of 1.35 fold in the grey scale value compared to control (Fig. 3c; *p* < 0.05). The PD flies exposed to 10, 20, 30 and 40 µM of kaempferol showed a dose dependent significant decrease of 1.03 1.08, 1.13 and 1.17 folds, respectively, in the grey scale values compared to unexposed PD flies (Fig. 3c; *p* < 0.05). The control flies exposed to 10, 20, 30 and 40 µM of kaempferol showed no significant difference in the grey scale values compared to unexposed control flies (Fig. 3c; *p* < 0.05). The PD flies exposed to 10^–3^ M of L-dopa showed a significant decrease of 1.14 fold in the grey scale value compared to PD flies (Fig. 3c; *p* < 0.05).

**Figure 4:**
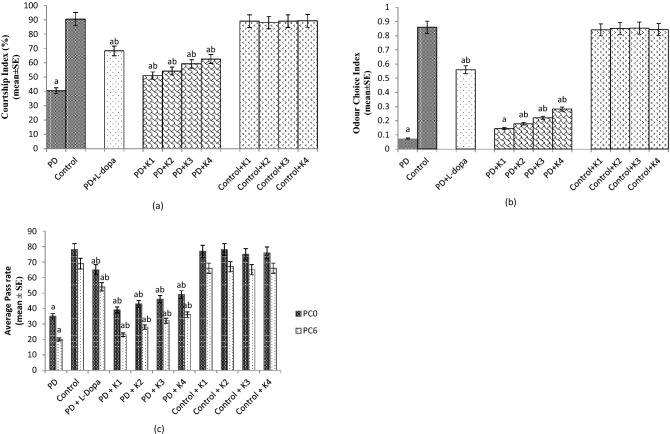
Effect of Kaempferol on Courtship Index (CI) (**a**), Odour Choice Index (OCI) (**b**) and Aversive Phototaxis Suppression Assay (APS assay) (**c**). The flies were allowed to feed on the diet supplemented with Kaempferol for 24 days. The values are the mean of 5 assays.[^a^significant with respect to control, *p* < 0.05; ^b^significant with respect to PD flies; L-Dopa: 10^−3^ M; K = Kaempferol; K1 = 10 μM; K2 = 20 μM; K3 = 30 μM; K4 = 40 μM; PD: PD flies].

The results obtained for courtship index showed a significant reduction of 2.25 fold in the CI of PD flies compared to control flies (Fig. 4a; *p* < 0.05). The PD flies exposed to 10, 20, 30 and 40 µM of kaempferol results in a significant dose dependent increase of 1.27, 1.35,1.47 and 1.57 folds respectively, in the courtship index compared to unexposed PD flies (Fig. 4a; *p* < 0.05). The PD flies exposed to 10^–3^ M of L-Dopa showed a significant increase of 1.70 fold in CI compared to unexposed PD flies (Fig. 4a; *p* < 0.05). The control flies exposed to 10, 20, 30 and 40 µM of kaempferol showed no significant difference in activity of CI compared to unexposed control flies (Fig. 4a; *p* < 0.05).

The odour choice index (OCI) of 24 days old PD flies showed a significant reduction of 12.28 fold compared to control flies (Fig. 4b; *p* < 0.05). The exposure of PD flies to 20, 30 and 40 µM of kaempferol showed a dose dependent significant increase of 2.42, 3.14 and 4 folds, respectively, in the OCI compared to unexposed PD flies (Fig. 4b; *p* < 0.05). The PD flies exposed to 10^–3^ M of L-Dopa showed a significant increase of eightfold in OCI compared to unexposed PD flies (Fig. 4b; *p* < 0.05). PD flies exposed to 10 μM of kaempferol showed no significant increase in the OCI compared to unexposed PD flies (Fig. 4b; *p* < 0.05). The control flies exposed to 10, 20, 30 and 40 µM of kaempferol showed no significant difference in OCI compared to unexposed control flies (Fig. 4b; *p* < 0.05).

A significant improvement in the memory was observed in the PD flies exposed to 10, 20, 30 and 40 µM of kaempferol, evaluated through aversive phototaxic suppression assay (Fig. 4c; *p* < 0.05). Likewise, exposure of PD flies to 10^–3^ M of L-Dopa showed a delay in the memory loss compared to unexposed PD flies (Fig. 4c; *p* < 0.05).

**Figure 5:**
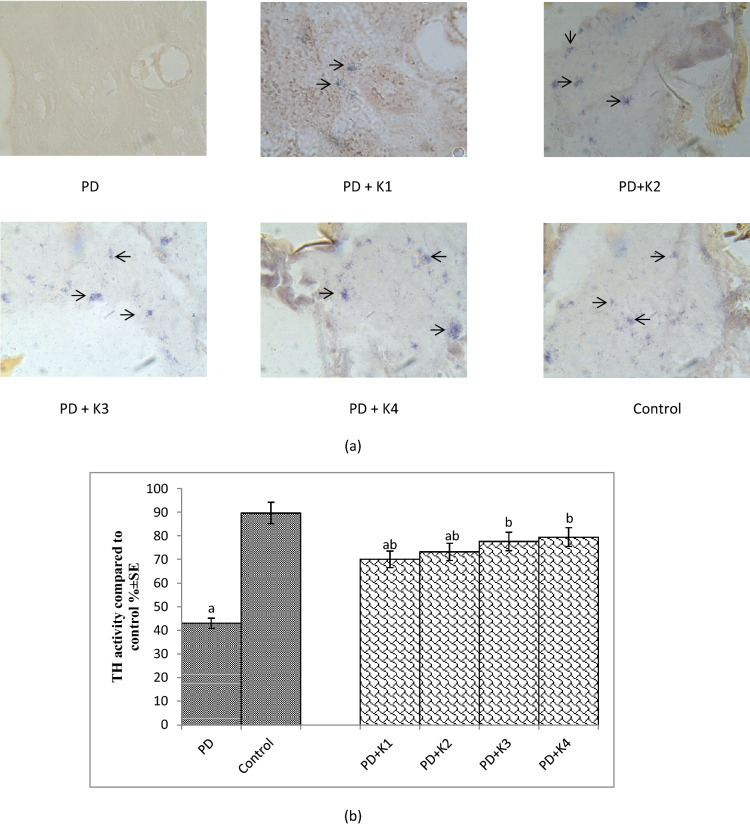
Tyrosine hydroxylase immunostaining performed on the brain section (**a**), quantification of Tyrosine hydroxylase on the brain section (**b**) of flies after 24 days of the exposure PD, L-Dopa: 10^−3^ M; K = Kaempferol; K1 = 10 μM; K2 = 20 μM; K3 = 30 μM; K4 = 40 μM; PD: PD flies].

The results obtained for tyrosine hydroxylase immunostaining are shown in Fig. 5a and the same has been quantified in Fig. 5b.The PD flies showed a marked age dependent reduction in the activity of tyrosine hydroxylase compared to control (Fig. 5a). The exposure of PD flies to different doses of kaempferol showed an increased immunoreactivity in comparison to unexposed PD flies (Fig. 5a). The PD flies showed a reduction of 2.07 fold in the TH^+^ cells (Fig. 5b; *p* < 0.05) compared to control flies. The PD flies exposed to 10, 20, 30 and 40 µM of kaempferol showed a significant dose dependent increase of 1.62, 1.70, 1.80 and 1.84 folds, respectively, in TH^+^cells compared to unexposed PD flies (Fig. 5b; *p* < 0.05).

**Figure 6:**
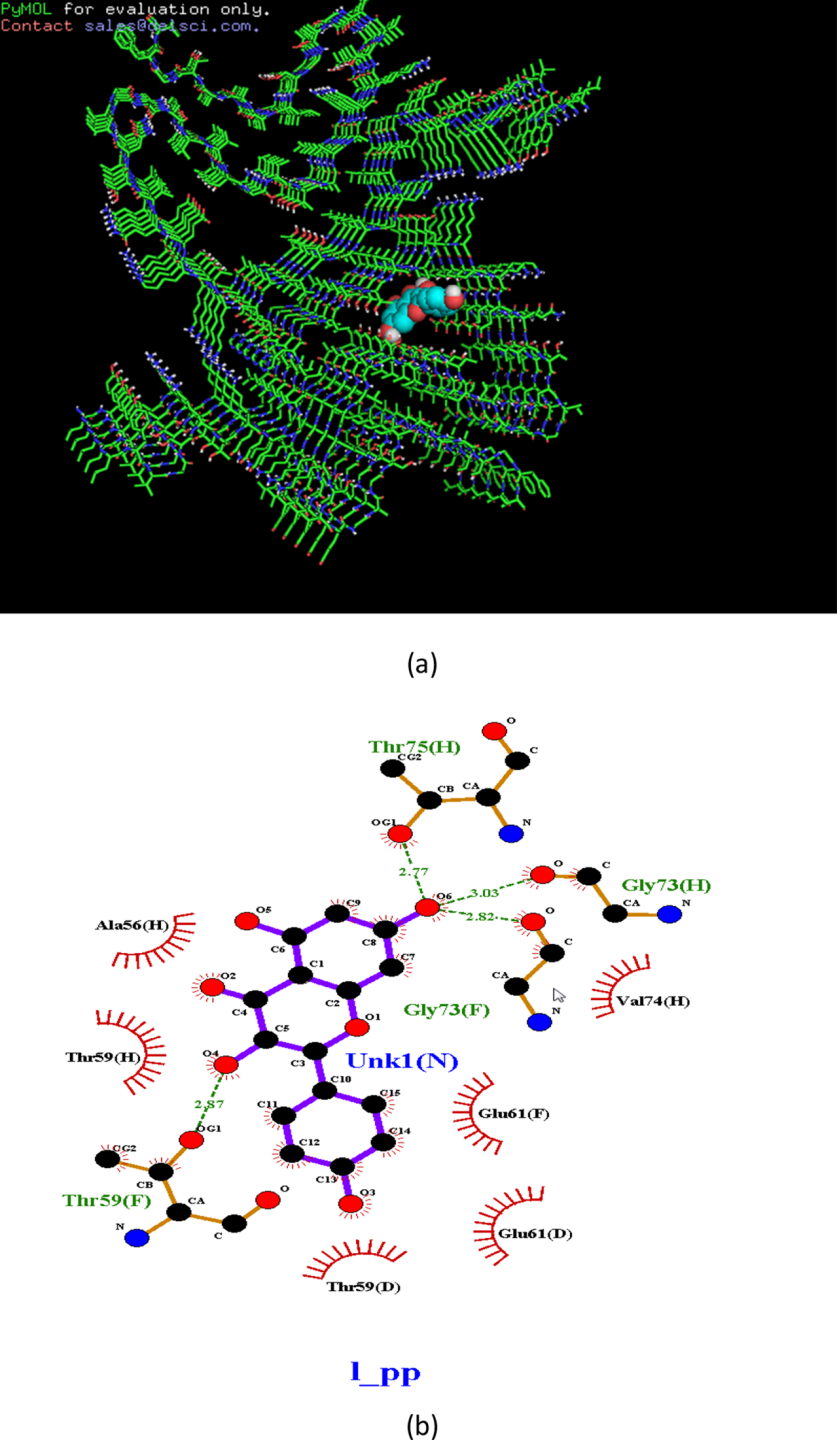
Binding site of kaempferol on the alpha synuclein protein showing the presence of hydrogen bond formed with threonine (**a**) and (**b**).

Ligplot analyses results showed 2D representation of ligand–protein interactions between alpha-synuclein and kaempferol (Fig. 6). Hydrogen bond forming residues are shown in green lines with hydrogen bonds shown as dotted lines and residues interacting by hydrophobic interactions are represented as red lines (Fig. 6).

## Discussion

The results of the present study reveal that the kaempferol is potent in reducing the oxidative stress, cognitive dysfunction, and prevents the loss of dopaminergic neurons. Although, L-Dopa is considered as the most effective drug to treat PD symptoms but its excessive use is associated with fluctuations in motor response and dyskinesia^13–15^. As oxidative stress is considered to be involved in the pathogenesis of PD hence any agent that reduced the oxidative stress in the brain may be use as a possible therapeutic agent^13^. The available medical treatments and operative procedures for PD only provide symptomatic relief^16^. Hence, it becomes necessary to explore new pharmacological agents based on natural plant products with less or no side effects for the protection against the pathogenesis of PD. Natural antioxidants such as phenols, flavonoids and tannins play an important role in reducing the oxidative stress by scavenging free radicals and reactive oxygen species thereby preventing the protein, lipid and DNA damage^17,18^.The autopsies of the brain of PD patients have revealed that oxidative stress is responsible for lipid, proteins and DNA damage along with decreased in the activity of SOD, catalase and glutathione levels^19–21^. Our present study on PD flies has also confirmed the same trend. The PD flies exposed to kaempferol showed a dose dependent increase in the scavenging of superoxide as well as free radicals. PD flies showed a decrease in the NPSH content. The PD flies exposed to various doses of kaempferol showed an increase in the NPSH content compared to unexposed PD flies. Glutathione-S-Transferase (GSTs) are phase II family of antioxidant enzymes that plays a vital role in the detoxification process during a condition of oxidative damage^22^. In our present study the PD flies showed an increase activity in GST activity but the PD flies exposed to kaempferol showed a dose dependent decrease in the GST activity. Glutathione (GSH) is synthesized by glycine, cysteine and glutamate and acts as a vital factor in the reduction of hydroperoxides, the quenching of free radicals, and the detoxification of xenobiotics^23^. Superoxide dismutases (SODs) are a group of metalloenzymes catalysing the dismutation of superoxide anion free radical (O_2_^−^) into molecular oxygen and hydrogen peroxide (H_2_O_2_)^24^. In our study the PD flies showed an increase in the SOD activity, however the PD flies exposed to kaempferol showed a dose dependent decrease in the activity of SOD. Catalase is responsible for breaking H_2_O_2_ to water and O_2_. The activity of CAT was higher in PD flies indicating the increased production of H_2_O_2,_ but the CAT activity was found to be decreased in a dose dependent manner in the PD flies exposed to various doses of kaempferol. The increase in TBARS and PC content in our study also supports the generation of free radicals that damage lipid membranes and protein. TBARS and protein oxidation are the reliable markers of oxidative stress. Hence, restoration of these markers towards the normal range gives an indication of the reduction in oxidative stress. A significant dose dependent decrease in the TBARS and PC content in the PD flies exposed to various doses of kaempferol supports its scavenging potential. Caspases are highly conserved cysteine proteases which play role in the regulation of apoptosis^25^. Drice and Dronc are orthologs of Caspase-3 and Caspase-9, respectively, in *Drosophila*^26^. Acridine orange is a vital dye that specifically stains cells undergoing apoptosis in *Drosophila melanogaster*^27^. It does not specifically stain the cells dying by oxygen starvation or necrosis^28^. The cells undergoing apoptosis will show bright fluorescence under fluorescent microscopy. A more intensity of fluorescence brain would possess a higher grey scale values compared to a less intensity fluorescing brain. Cell death and neuronal dysfunction are hallmarks of age related neurodegenerative disorders^29^. In our present study the PD flies exposed to kaempferol showed a dose dependent decrease in the activity of Caspase-9, 3 and grey scale values suggesting the anti-apoptotic role of kaempferol. Our earlier studies with natural plant products/extract have also suggested the anti-apoptotic role by performing the same assays^30,31^. Our results obtained for DPPH and superoxide anions scavenging assays also support the antioxidant potential of kaempferol that could be helpful in reducing the oxidative stress. Ligplot analyses between alpha-synuclein and kaempferol showed two residues Thr59 and Ala56 which are proved to hold the terminal ring to be cleaved in the catalytic reaction by forming a hydrophobic patch are observed to be present. The Val74 was also observed to form forming hydrophobic interactions. It was observed that a hydrophobic patch comprising of Thr59 and Glu61 hold the terminal ring of kaempferol. Ligplot analyses are especially useful in knowing the hydrophobic interaction pattern. It has been reported that α-synuclein enhances the fragmentation of mitochondria, disrupt mitochondrial membrane potential and inhibit autophagy^32^. Mitochondrial damage leads to the increased production of ROS enhancing the aggregation of α-synuclein and finally leading to the formation of Lewy bodies^33^. These events lead to the loss of dopaminergic neurons which results in the decrease of dopamine content in the brain^7^. L-Dopa is converted into dopamine by the action of tyrosine hydroxylase and is also a rate limiting step. In our present study the PD flies showed less TH immunopositive cells. The pathophysiology of PD involves the dysregulation of the nigrostriatal dopaminergic system, with a decrease in TH activity in the striatum of PD patients and in animal models^34^. The PD flies exposed to various doses of kaempferol showed a dose dependent increase in the TH immunopositive cells. The protective role of kaempferol is due to the reduction in the oxidative stress in the brain of PD flies on its exposure. Kaempferol is potent in scavenging free radicals, thereby leading to the reduction in oxidative stress and protects the neurons.

Olfactory dysfunction is very frequent in PD and has been reported in number of studies performed on PD patients^35–37^. Post-mortem studies performed on PD patients revealed that due to the formation of Lewy bodies in the olfactory bulb and in other regions of the brain such as the anterior olfactory nucleus, the piri-form cortex, the amygdaldoid complex, the entortinal cortex and the hippocampal may lead to the olfactory dysfunction^38,39^. However, correlation has also been suggested between the death of dopaminergic neurons and olfactory dysfunction^40^. Invertebrate brain though simpler but is capable of directing complex behaviors, learning and memory. In *Drosophila*, the mushroom body is a collection of small neurons called Kenyon cells. These cells are situated on top of the calyx, the site of sensory input^41,42^. Mushroom body is important for learning, memory, olfaction and spatial navigation^43^. In our present study with the transgenic flies expressing human alpha synuclein in the neurons and the formation of LBs may also lead to the damage of neurons of the mushroom bodies and hence these flies exhibit the loss of olfaction and memory.

**Figure 7:**
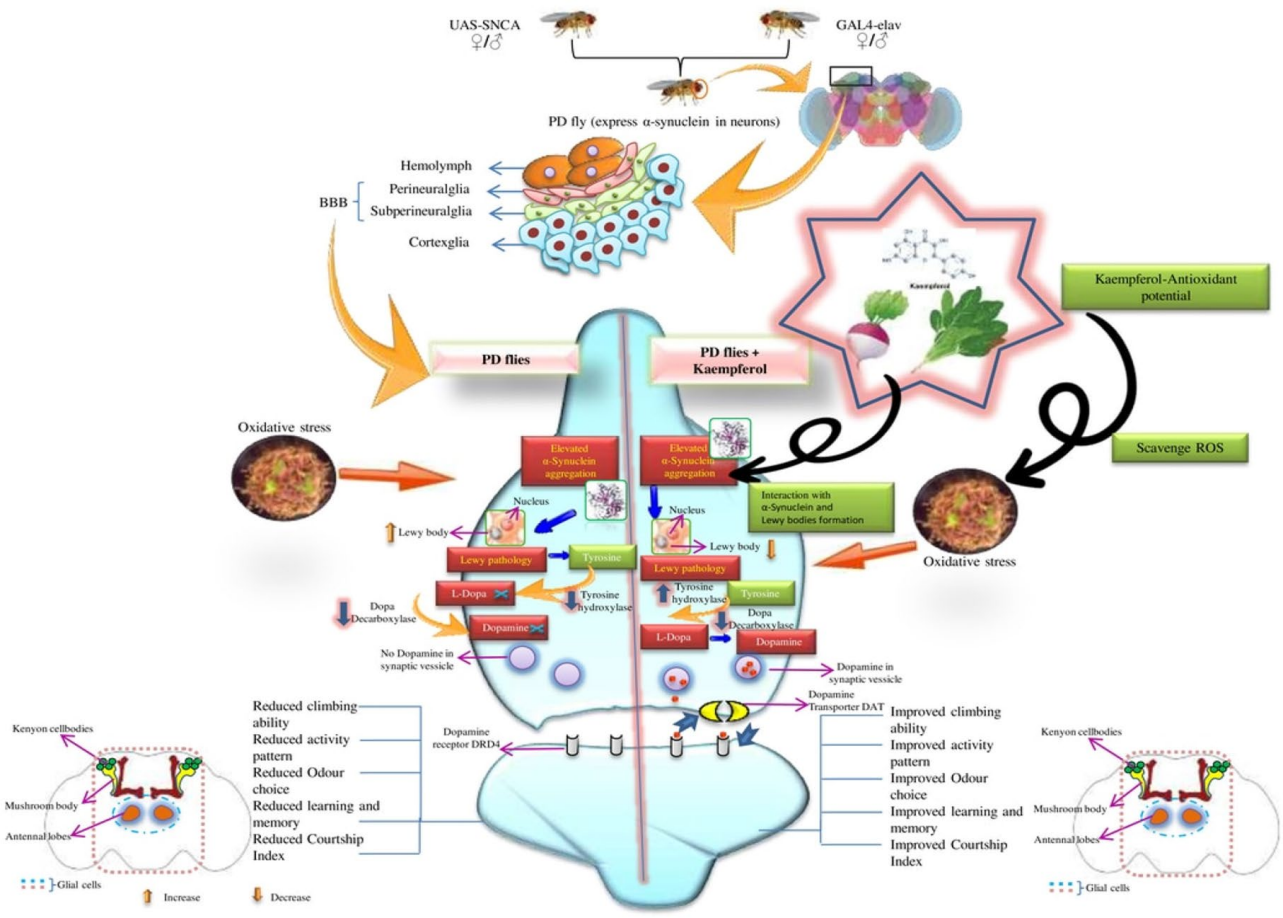
Possible mechanism of free radical scavenging by Kaempferol which it binds to α-synuclein thereby inhibiting Lewy body formation and preventing α-synuclein aggregation.

In our present study the PD flies showed a reduction in the odour choice index however the PD flies exposed to various doses of kaempferol showed a dose dependent increase in the OCI. The present model express α-synuclein panneurally hence its expression and the formation of Lewy bodies may also affects the region of the *Drosophila* brain that controls the olfaction i.e. mushroom body. The formation of Lewy bodies affects the neurons of the mushroom body and thus flies exhibits the loss of olfaction. The exposure of kaempferol reduced the oxidative stress in the neurons as a result the damage to the neurons is reduced and therefore the PD flies showed an increase in the OCI. Sexual behavior is a functioning of complex rituals involving autonomic, sensory and motor systems^44^. It has been reported that PD may increase sexual dysfunction by up to six times^45^. There are evidences which indicate that the central dopaminergic system has a major role in the control of sexual function both in animals and humans. Dopamine depletion in PD has been associated with the impairment of desire and arousal^46^. In *Drosophila* courtship ritual involves orientation of the male towards the female, serenading the female with a species-specific love song (wing vibration), licking the females genitalia, and attempting copulation^5^. Since the courtship involves many neural and motor elements, it might be affected by the expression of α-synuclein. A decline in the behavioral response has been reported in the transgenic flies expressing A30P α-Synuclein in the brain^7^. In our present study the PD flies showed a significant decrease in the courtship index. The PD flies exposed to various doses of kaempferol showed a dose dependent significant increase in the CI. The loss of neurons can also lead to the memory loss^47^. In our study the PD flies showed the loss of memory as measured by performing aversive phototaxis assay. The natural plant products have been shown to improve the memory functions in various experimental models^48–50^. Based on the results obtained in our present study the possible mode of protection by kaempferol has been depicted in Fig. 7. The Fig. 7 states that the expression of alpha synuclein in the neurons leads to the formation of Lewy bodies which selectively damage the dopaminergic neurons. The formation of Lewy bodies and the damage of the neurons lead to a state of oxidative stress which further enhance the neurodegeneration. Due to the loss of dopaminergic neurons, dopamine is not available and the flies exhibit cognitive impairments. The PD flies exposed to kaempferol showed the reduction in the oxidative stress as a result the neurons are not damaged hence the dopamine is available and PD flies exhibit improvement in cognitive impairments. This is evident by the increased activity of tyrosine hydroxylase in the PD flies exposed to various dose of kaempferol. Kaempferol is able to cross the blood brain barrier (BBB)^12^ and the BBB of *Drosophila* is not as that complex compared to humans. After crossing the BBB, kaempferol due to its antioxidant and free radical scavenging potential exhibit a protective effect.

In our earlier study with transgenic Alzheimer’s disease model flies kaempferol was found to reduce the oxidative stress markers and associated cognitive dysfunction functions^51^. Bioactive compounds from medicinal plants have gained interest due to their little or no toxicity. The neuroprotection of the kaempferol is due to its antioxidant property. Hence, it is concluded from our present study that kaempferol is also potent in reducing the PD symptoms being mimicked in transgenic flies and can be used as possible therapeutic agent against neurodegenerative disorders.

## Materials and methods

### Drosophila stocks

Transgenic fly lines that express wild-type human synuclein (h-αS) under UAS control in neurons “.w.*.;P{w. + mC. = UAS–Hsap/SNCA.F}’’5B and GAL4 “w.*.;P{w. + mC. = GAL4- elavL}’’3. were obtained from Bloomington Drosophila Stock Centre (Indiana University, Bloomington, IN). When the males of UAS (Upstream Activation Sequence)-Hsap/SNCA.F strains were crossed with the females of GAL4-elav. L (Vice-versa), the progeny expressed human αS in the neurons^52^.

### Drosophila culture and crosses

The flies were cultured on standard *Drosophila* food containing agar, corn meal, sugar and yeast at 25 °C (24 ± 1)^53^. Crosses were set up as described in our earlier published work^54^. The PD flies were allowed to feed separately on different doses of kaempferol mixed in the diet. Kaempferol was added in the diet at final concentration of 10, 20, 30 and 40 µM. The PD flies were also exposed to 10^–3^ M of L-dopa. The UAS Hsap/SNCA.F act as a control. The control flies were separately allowed to feed on the selected doses of kaempferol.

### Antioxidative assays

Two assays were performed for estimating the antioxidative potential of kaempferol for the doses selected in our present study:

### Diphenyl-picrylhydrazyl (DPPH) free radical scavenging

For estimating free radical scavenging potential of the kaempferol, DPPH method as described by Wongsawatkul et al.^55^ was used in our present study. When DPPH (a stable purple colour) react with an antioxidant, it is reduced to yield a light yellow coloured diphenylpicrylhydrazine. The reaction mixture consisted of 500 µl of kaempferol and 250 µl of DPPH (0.3 mM). The reaction mixture was shaken vigorously and allowed to stand at room temperature in the dark for 25 min. The OD was read at 518 nm and the percentage of radical scavenging activity was calculated by the following equation:

$$\% {\text{ Radical}}\;{\text{scavenging}} = \left( {{1} - {\text{Absorbance}}\;{\text{of}}\;{\text{sample}}/{\text{Absorbance}}\;{\text{of}}\;{\text{control}}} \right) \times {1}00$$

### Superoxide anion scavenging assay

The inhibition of Nitrobluetetrazolium (NBT) reduction by phenazinemethosulphate (PMS) generated O_2_^−^ was used to determine the superoxide anion scavenging activity of the kaempferol^56^. The reaction mixture consisted of 75 µl of each concentration of kaempferol, 750 µl of TrisHCl (100 mM; pH 7.4); 187 µl of NBT (300 µM), 187 µl of NADH (936 µM). The reaction was initiated by adding phenazinemethosulphate (PMS) (120 µM). The reaction mixture was incubated at 25 ºC for 5 min and the OD was read at 560 nm and the degree of scavenging was calculated by the following equation:

$${\text{Scavenging}}(\% ) = \left[ {\frac{{{\text{OD}}\;{\text{control}} - {\text{OD }}\;{\text{sample}}}}{{{\text{OD }}\;{\text{control}}}}} \right] \times { }100$$

### Drosophila activity pattern

From the 12th day the activity of flies (males) in all treated groups was analyzed by using Drosophila Activity Monitor (TriTek, USA). The activity was recorded every hour for a total of 288 h and the data was analyzed by Actogram J software. The results were presented as activity pattern and chi-square periodogram^57^.

### Retinal degeneration analysis by SEM

Heads of 24 days old flies (5/treatment; 5 replicates/group) were fixed overnight in an ice-cold solution Karnovsky’s fixative (2.5% glutaraldehyde, 2.0% formaldehyde in 0.1 M phosphate buffer, pH 7.3) for 1 h. After rinsing in buffer, heads were post-fixed for 1 h in 0.5% osmium tetraoxide and processed according to the procedure as described by Chen et al.^58^. Samples were then imaged using JEOL SEM (JSM-6510LV).

### Drosophila climbing assay

The climbing assay was performed as described by Pendleton et al.^59^_._ Ten flies were placed in an empty glass vial (10.5 cm × 2.5 cm). A horizontal line was drawn 8 cm above the bottom of the vial. After the flies had acclimated for 10 min at room temperature, both controls and treated groups were assayed at random, to a total of 10 trails for each. The mean values were calculated and then averaged and a group mean and standard error were obtained. All behavioral studies were performed at 25 °C under standard lighting conditions.

### Preparation of homogenate for biochemical parameters

Fly heads from each group were taken (50 heads/group; five replicates/group) and the homogenate was prepared in 0.1 M phosphate buffer for the biochemical parameters.

### Lipid peroxidation assay

Lipid peroxidation was measured according to the method described by Ohkawa et al.^60^. The reaction mixture consisted of 5 µl of 10 mM butyl-hydroxytoluene, 200 µl of 0.67% thiobarbituric acid, 600 µl of 1% O-phosphoric acid, 105 µl of distilled water and 90 µl of supernatant. The resultant mixture was incubated at 90 °C for 45 min and the OD was measured at 535 nm. The results were expressed as µmol of TBARS formed/h/g tissue.

### Estimation of protein carbonyl content (PCC)

The PC content was estimated according to the protocol described by Hawkins et al.^61^. The brain homogenate was diluted to a protein concentration of approx 1 mg/ml. About 250 µl of each diluted homogenate was taken in an eppendorf centrifuge tubes separately. To it 250 µl of 10 mM 2, 4-dinitrophenyl hydrazine (dissolved in 2.5 M HCl) was added, vortexed and kept in dark for 20 min. About 125 µl of 50% (w/v) trichloroacetic acid (TCA) was added, mixed thoroughly and incubated at − 20 °C for 15 min. The tubes were then centrifuged at 4 °C for 10 min at 9000 rpm. The supernatant was discarded and the pellet obtained was washed twice by ice cold ethanol: ethyl acetate (1:1). Finally, the pellets were re-dissolved in 1 ml of 6 M guanidine hydrochloride and the absorbance was read at 370 nm.

### Estimation of monoamine oxidase (MAO)

The method described by Pine et al.^62^ was used to estimate the monoamine oxidase activity. The assay mixture consisted of 400 µl of 0.1 M phosphate buffer (pH 7.4), 130 µl of distilled water, 100 µl of benzylamine hydrochloride and 200 µl of brain homogenate. The assay mixture was incubated for 30 min at room temperature and then 1 ml of 10% perchloric acid was added and centrifuged at 2000 rpm for 10 min. The OD was taken at 280 nm.

### Estimation of glutathione (GSH) content

The GSH content was estimated colorimetrically using Ellman’s reagent (DTNB) according to the procedure described by Jollow et al.^63^. The supernatant was precipitated with 4% sulphosalicyclic acid in the ratio of 1:1. The samples were kept at 4 °C for 1 h and then subjected to centrifugation at 5000 rpm for 10 min at 4 °C. The assay mixture consisted of 550 µl of 0.1 M phosphate buffer, 100 µl of supernatant and 100 µl of DTNB. The OD was read at 412 nm and the results were expressed as µ moles of GSH/gram tissue.

### Estimation of glutathione-S-transferase (GST) activity

The GST activity was estimated by the method of Habig et al.^64^.The reaction mixture consisted of 500 µl of 0.1 M phosphate buffer, 150 µl of 10 mM 1 chloro 2,4 dinitro benzene (CDNB), 200 µl of 10 mM reduced glutathione and 50 µl of supernatant. The OD was taken at 340 nm and the enzyme activity was expressed as µ moles of CDNB conjugates/min/mg protein.

### Estimation of catalase (CAT) activity

The catalase activity was estimated according to the method of Beers and Sizer^65^ by kinetic method where rate of dismutation of H_2_O_2_ to water and molecular oxygen is proportional to the concentration of catalase in the sample. The reaction mixture consisted of 650 μl of 0.1 M phosphate buffer, 333 μl of H_2_O_2_ (0.05 M) and 17 μl of sample. A decrease in OD was measured for 2 min, at 30 s interval at 240 nm. The activity of catalase was calculated and expressed as μmoles of H_2_O_2_ consumed/min/mg protein.

### Estimation of superoxide dismutase activity (SOD) activity

The assay was performed according to the method described by Marklund and Marklund^66^.The reaction mixture consisted of 17 μl of sample and 950 μl of 0.1 M phosphate buffer. The reaction was initiated by adding pyrogallol. An increase in OD was noted at 420 nm for 3 min at 30 s interval and the results were expressed as units/mg protein.

### Spectrophotometric assay for caspase-9 (Dronc) and caspase-3 (Drice) activities

The assay was performed according to the manufacturer protocol with some modification (Bio-Vision, CA, USA). After isolating the brain, a treatment of collagenase (0.5 mg/ml in PBS; pH 7.4) was given before performing the assay for 10 min .The assay was based on spectrophotometric detection of the chromophore *p*-nitroanilide (pNA) obtained after specific action of caspase-3 and caspase-9 on tetrapeptide substrates, DEVD-pNA and IETD-pNA, respectively. The assay mixture consisted of 50 µl of fly homogenate and 50 µl of chilled cell lysis buffer incubated on ice for 10 min. After incubation, 50 µl of 2X reaction buffer (containing 10 mM DTT) with 200 µM substrate (DEVD-pNA for Drice, and IETD-pNA for Dronc) was added and incubated at 37 ºC for 1.5 h. The reaction was quantified at 405 nm.

### Analysis of cell death in the Drosophila brain

The cell death in the *Drosophila* brain was analyzed as per the method described by Mitchell and Staveley^67^. After 24 days of exposure of PD flies to various doses of kaempferol, the brain were isolated in Ringer’s solution under stereozoom microscope. Flies (20/treatment; 5 replicates/group) were placed in 70% ethanol in a 2 ml microcentrifuge tube for a minute. After removing the Ringer’s solution, about 100 µl of freshly prepared acridine orange (5 µg/ml) was added for 5 min. Each brain was rinsed by Ringer’s solution, immediately viewed and photographed through fluorescent microscope (OPTIKA, Italy). The image analysis program Image J (available online at https://rsb.info.nih.gov/ij/) was used to analyze the grey scale values for each brain.

### Courtship assay

The assay was performed by the method of Nichols et al.^68^. Newly eclosed virgin male and female flies were separated and kept in different diet vials for 24 days at 25 °C under 12 h light/dark. On the day of experiment one pair of flies was transferred into a mating chamber and observed. Observations were made till successful copulation noting the time of each behaviour (orientation, male song, chasing and licking) and the total time of courtship behaviours until mating. The courtship index (C.I.) was calculated by dividing the time of courtship by the total time until copulation.

### Odour choice index

The assay was performed by the method of Simonnet et al.^69^. The test flies were starved for 16–18 h at 25 °C before the experiment. Two small filter paper dipped in propionic acid and octanol, respectively, were kept in the two tubes of the Y maze. First the experiment was performed by introducing the newly enclosed flies into the Y maze and then the same was repeated with 24 days old flies from all the groups. The number of flies entering both the tubes were counted. Five replicates per group and 20 flies per group were used in the assay. The odour choice index was calculated as: Number of flies in tube1- Number of flies in tube 2)/ total number of flies.

### Aversive phototaxis suppression assay (APS assay)

This assay makes use of positive phototactic behavior in flies to associate light with aversive stimuli (Quinine in this experiment) and was performed according to described by Ali et al.^70^ 24 day old male flies were selected for the experiment from each group (3 replicates/group). First the flies were trained to develop a short term memory against 1 μM of quinine in a T-maze. Immediately after the training the learning behavior of flies for each group were recorded as PC0 (0 h post conditioning). After 6 h the experiment was repeated and the readings were recorded as PC6 (6 h post conditioning).

### Immunohistochemistry

The fly heads were isolated and the paraffin sections were prepared according to the procedure described by Palladino et al.^71^ The sections were deparaffinized and rehydrated. The slides were blocked in 8% Bovine Serum Albumin (BSA) for 2.5 h. Then the slides were washed with phosphate buffer saline (pH 7.2) containing 2% BSA for 5 min. After washing, the slides were incubated with primary antibody (anti-tyrosine hydroxylase, Merck; 1:1000) in a humidified chamber for 12 h at 4 °C. The slides were then washed with PBS containing 2% BSA for 5 min and incubated with secondary antibody (Goat anti-Rabbit alkaline phosphatase, Santacruz, Biotechnology, USA; 1:100) at room temperature for 2 h. The final wash was given by PBS containing 2% BSA for 5 min. BCIP-NBT was used as a chromogenic substrate which interacts with secondary antibody to produce blue coloured product. The slides were then mounted in DPX and observed under the microscope.

### Molecular docking

The molecular docking was performed to study the interaction between alpha synuclein and kaempferol. It was performed by using PyMOL and Ligplot. PyMOL is a free cross-platform molecular graphics system provides most of the capabilities and performance of traditional molecular graphics packages written in C or Fortran. Ligplot is an essential tool to understand hydrophobic interactions as well as hydrogen bonding pattern. The structure of alpha synuclein was taken from the RCSB protein data bank (https://www.rcsb.org/pdb/home/home.do) and of kaempferol was obtained from the PubChem (https://pubchem.ncbi.nlm.nih.gov). The docking was done keeping the protein constant and allowing the flavonoid molecule to explore its conformational positions. Visualisation of the protein–flavonoid interaction was done by PyRx (Python Prescription).PyRx is open source software to perform virtual screening, using the combination of software Vina and AutoDock 4.2^13^.

### Statistical analysis

The data was subjected to statistical analysis through one way analysis of variance (ANOVA) followed by posthocTukey test using GraphPad Prism software (version 5.0). The level of significance was kept at *p* < 0.05.The results were expressed as mean ± SEM.

## Supplementary information

Supplementary Information.

## Abbreviations

PD: Parkinson’s disease
TH: Tyrosine hydroxylase
DOPA: 3,4-Dihydroxy phenylalanine
UPS: Ubiquitin-proteasomal system
ALP: Autophagy-lysosomal pathway
MPTP: 1-Methyl-4-phenyl-1,2,3,6-tetrahydropyridine
6-OHDA: 6-Hydroxydopamine
ROS: Reactive oxygen species
NOS: Nitric oxide synthase
RNS: Reactive nitrogen species
MAO: Monoamine oxidase
OCI: Odour choice index
CI: Courtship index
GSH: Glutathione
GST: Glutathione-S-transferase
LPO: Lipid peroxidation
PC: Protein carbonyl
MAO: Monoamine oxidase
SOD: Superoxide dismutase
CAT: Catalase
TH: Tyrosine hydroxylase
BCIP: 5-Bromo-4-chloro-3′-indolyphosphate *p*-toluidine
NBT: Nitro blue tetrazolium chloride
TCA: Trichloroacetic acid
APS: Aversive phototaxis suppression
PyRx: Python prescription
PBS: Phosphate buffer saline
