# Supplementary material for: Effect of kaempferol on the transgenic Drosophila model of Parkinson’s disease
Source: Sci Rep. 2020 Aug 14;10:13793. doi: 10.1038/s41598-020-70236-2 (PMC7429503; doi:10.1038/s41598-020-70236-2)
Supplement: Supplementary file 1 — Supplementary Information. [file 41598_2020_70236_MOESM1_ESM.docx]

**Effect of Kaempferol on the transgenic Drosophila model of Parkinson’s disease**

Rahul, Falaq Naz, Smita Jyoti and Yasir Hasan Siddique*

Drosophila Transgenic Laboratory, Section of Genetics, Department of Zoology, Faculty of Life Sciences, Aligarh Muslim University, Aligarh, Uttar Pradesh- 202002, India.

*Email: [yasir_hasansiddique@rediffmail.com](mailto:yasir_hasansiddique@rediffmail.com)

Ph.No: 0571-2700920-3447

Figure S1: Radical scavenging activity of kaempferol [K= Kaempferol; K1=10μM; K2=20 μM; K3=30 μM; K4=40 μM;  ^*^significant at p<0.05 compared to control]. Ascorbic acid was used as control.

Figure S2:Superoxide anion scavenging activity of kaempferol [K= Kaempferol; K1=10μM; K2=20 μM; K3=30 μM; K4=40 μM;^*^significant at p<0.05 compared to control]. Ascorbic acid was used as control.


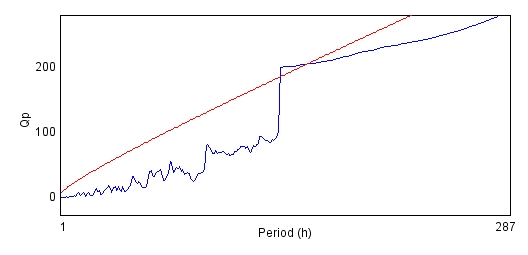

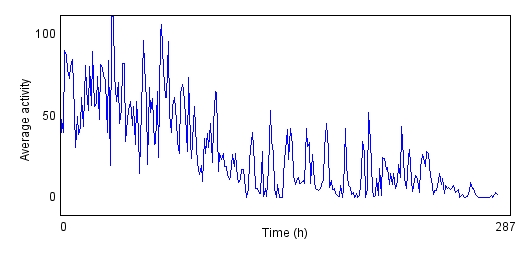


a b

Figure S3: (a) and (b) show the average activity pattern and chi-square periodogram, respectively, for the PD flies (𝑁 = 20).


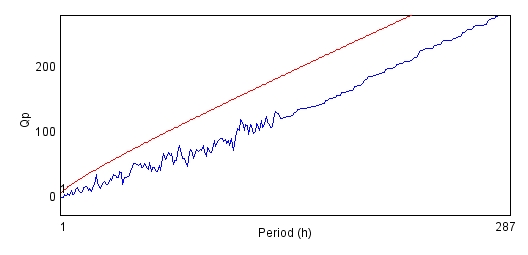

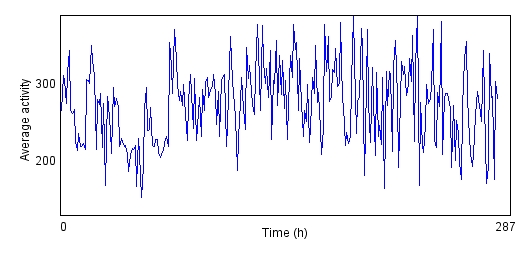


a b

Figure S4: (a) and (b) show the average activity pattern and chi-square periodogram, respectively, for the control flies (𝑁 = 20).


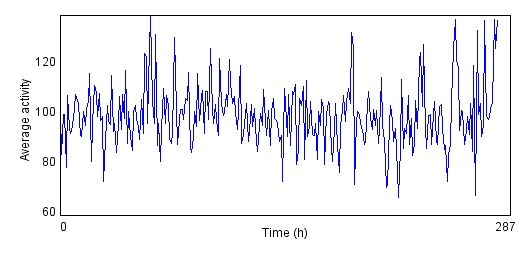

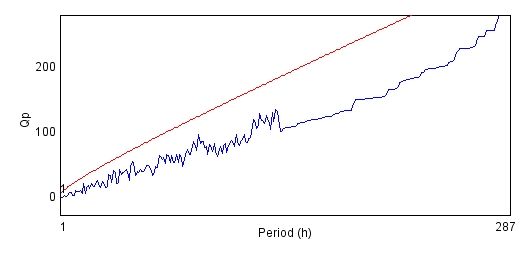


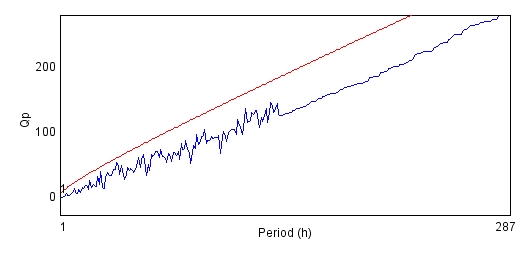

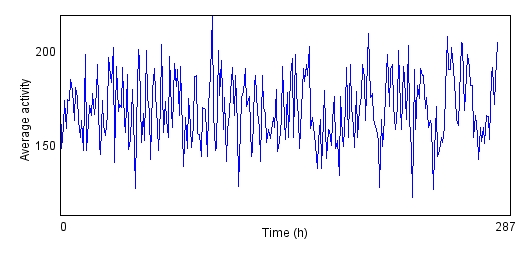

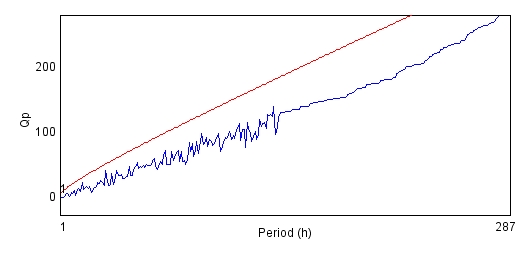

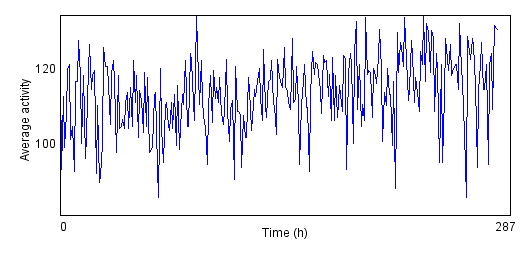


a b

a b

a b

Figure S6:(a) and (b) show the average activity pattern and chi-square periodogram, respectively, for the PD flies exposed to 20 𝜇M of kaempferol in diet (𝑁 = 20).

Figure S7: (a) and (b) show the average activity pattern and chi-square periodogram respectively, for the PD flies exposed to 30 𝜇M of kaempferol in diet (𝑁 = 20).

Figure S5: (a) and (b) show the average activity pattern and chi-square periodogram, respectively, for the PD flies exposed to 10 𝜇M of kaempferol in diet (𝑁 = 20).


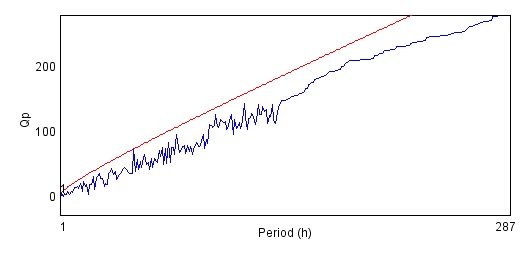

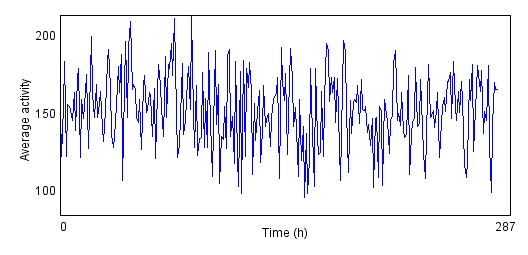


a b

Figure S8: (a) and (b) show the average activity pattern and chi-square periodogram respectively, for the PD flies exposed to 40 𝜇M of kaempferol in diet (𝑁 = 20).


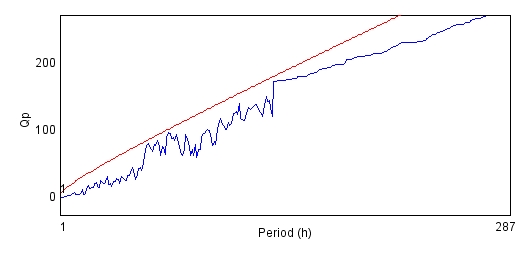

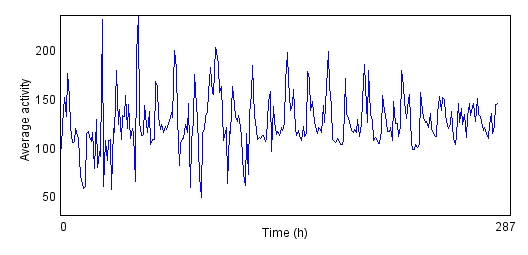


a b

Figure S9: (a) and (b) show the average activity pattern and chi-square periodogram respectively, for the control flies exposed to 10𝜇M of kaempferol in diet (𝑁 = 20).


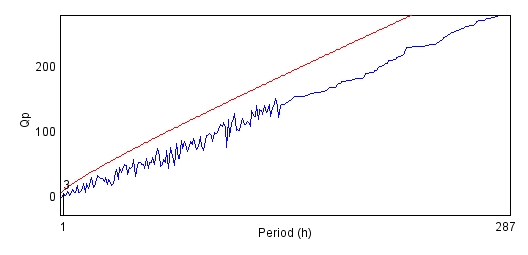

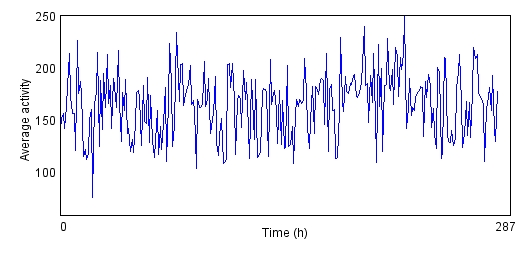


a b

Figure S10: (a) and (b) show the average activity pattern and chi-square periodogram, respectively, for the control flies exposed to 20 𝜇M of kaempferol in diet (𝑁 = 20).


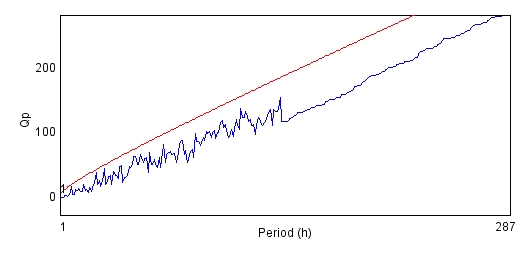

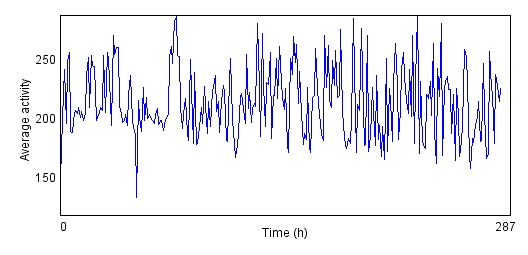


a b

Figure S11: (a) and (b) show the average activity pattern and chi-square periodogram, respectively, for the control flies exposed to 30 𝜇M of kaempferol in diet (𝑁 = 20).


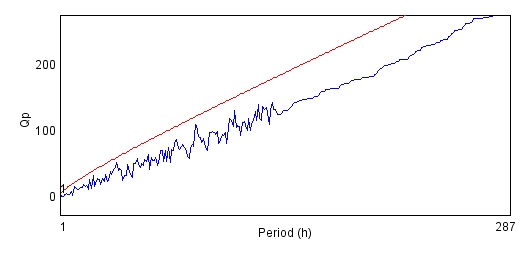

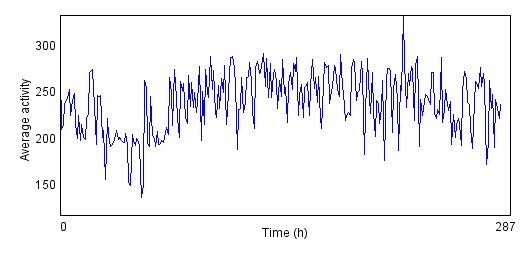


a b

Figure S12: (a) and (b) show the average activity pattern and chi-square periodogram, respectively, for the control flies exposed to 40 𝜇M of kaempferol in diet (𝑁 = 20).


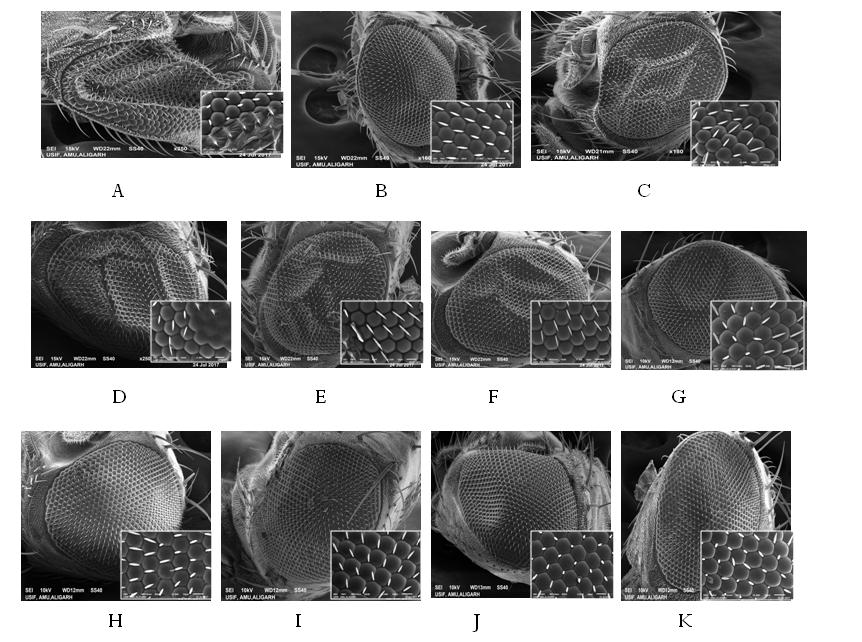

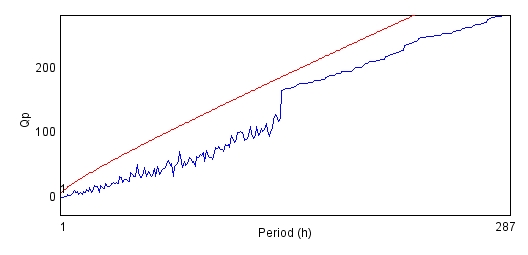

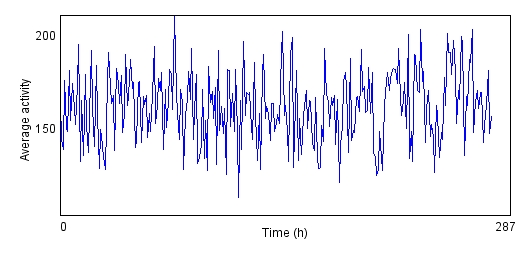


a b

Figure S 14.Effect of Kaempferol on the retinal degeneration in eyes of flies. The flies were allowed to feed on the diet supplemented with Kaempferol for 24 days and then assayed for retinal degeneration .A=PD, B= control, C= PD + L-dopa, D= PD +K1= 10𝜇M; E= PD +K2 =20 𝜇M; F= PD +K3= 30𝜇M; G= PD +K4= 40𝜇M; H= C +K1= 10𝜇M; I= C +K2 =20𝜇M; J= C +K3= 30𝜇M; K= C +K4= 40𝜇M

Figure S13:(a) and (b) show the average activity pattern and chi-square periodogram, respectively, for the PD flies exposed to 10^-3^ M of L-dopa in diet (𝑁 = 20).
